# Prevalence of self-reported dental pain and associated factors among eight- to ten-year-old Brazilian schoolchildren

**DOI:** 10.1371/journal.pone.0214990

**Published:** 2019-04-08

**Authors:** Pablo Silveira Santos, Paulo Antônio Martins-Júnior, Saul Martins Paiva, Daniele Klein, Fernanda Marques Torres, Angela Giacomin, Bruna Miroski Gonçalves, Andrea Cristina Konrath, Michele Bolan, Mariane Cardoso

**Affiliations:** 1 Postgraduate Program in Dentistry, Federal University of Santa Catarina, Florianópolis, Santa Catarina, Brazil; 2 Department of Pediatric Dentistry and Orthodontics, Federal University of Minas Gerais, Belo Horizonte, Minas Gerais, Brazil; 3 Department of Informatics and Statistics, Federal University of Santa Catarina, Florianópolis, Santa Catarina, Brazil; 4 Department of Pediatric Dentistry, Federal University of Santa Catarina, Florianópolis, Santa Catarina, Brazil; School of Dentistry, University of Sao Paulo, BRAZIL

## Abstract

**Aim:**

To assess the prevalence of self-reported dental pain and its association with sociodemographic, clinical and behavioral/psychosocial indicators among 8- to 10-year-old Brazilian schoolchildren.

**Design:**

A cross-sectional study was carried out with 1,589 eight- to ten-year-old children randomly selected from public schools of Florianopolis, Brazil. Self-reports of dental pain were collected through a single question as follows: “In the last month, how many times have you had pain in your teeth?” Caries experience was determined by the DMFT/dmft index and its clinical consequences by the PUFA/pufa index. Dental trauma, dental fluorosis and molar-incisor hypomineralization were assessed through the Andreasen, Dean and European Academy of Pediatric Dentistry classifications, respectively. Clinical signs of dental erosion were also observed. Sociodemographic indicators were obtained through a questionnaire answered by the children’s caregivers. Information about behavioral/psychosocial indicators was collected through questions from the Brazilian version of the Child Perception Questionnaire 8–10 years (CPQ_8–10_). Descriptive analysis, chi-square test, and hierarchically adjusted Poisson regression models were performed.

**Results:**

819 children (51.5%) reported episodes of dental pain in the last month prior to the study, whereas 55.6% (n = 509) were girls. The presence of dental pain was significantly associated with sex, trouble sleeping, difficulty eating, school absenteeism, difficulty with paying attention in class, difficulty doing homework, staying away from recreational activities, caries experience, PUFA/pufa index and ulceration (p<0.05).

**Conclusion:**

The prevalence of self-reported dental pain in 8- to 10- year-old Brazilian schoolchildren was high and was associated with sociodemographic, clinical and behavioral/psychosocial indicators.

## Introduction

Pain is a multidimensional phenomenon composed of physiological and psychological variables associated with current or potential tissue damage [1]. Dental pain is the most common symptom that compels patients to seek dental treatment and it has emotional, biologic and psychological meaning to patients [2].

The overall lifetime prevalence of dental pain among schoolchildren in developed countries ranges from 11.8% to 47.5% [3,4]. A prevalence of dental pain of 7.6% was observed in 8-year-old schoolchildren from England when only considered the previous month of data collection [3]. In contrast, 70% of 8 to 10-year-old schoolchildren from Cape Town, South Africa, and 25% of 8-year-old children from Sri Lanka had experienced dental pain in the previous two months of the surveys [5,6]. In addition, a prevalence of dental pain of 15.6% was observed in 8 to 9-year-old Brazilian schoolchildren, when considered the previous month of data collection [7].

Untreated dental caries and its clinical consequences are the most consistent clinical correlates of dental pain [8]. Among these consequences, clinical conditions such as untreated dental caries infecting pulpal tissue and the presence of abscesses have been associated with reports of dental pain [9]. Furthermore, other oral conditions such as dental trauma and oral ulcers can also cause pain and, consequently, exert negative effects on the daily living of children [10]. Besides that, studies have shown that school absenteeism, sleep disturbances, difficulty in chewing and difficulty in socialization are events commonly associated to dental pain in children and adolescents [11,12].

Despite the high prevalence of untreated dental caries among Brazilian schoolchildren, there has been a limited amount of high-quality research on the prevalence of dental pain among 8- to 10-year-old children. The majority of studies addressing dental pain were carried out with preschool children or with children already presenting complete permanent dentition. Further studies focusing on children with mixed dentition are needed, since it is a crucial phase for the proper development of the permanent dentition. In this phase, the presence of dental pain may be an indicator of damage to dental occlusion (e.g.: premature tooth loss) and future need for a long-term dental treatment. In addition, the ages of 8–10 years are of pivotal importance in the psychological development, and dental pain can negatively affect a child’s emotional and phycosocial well-being [10].

Therefore, the purpose of the present study was to assess the prevalence of self-reported dental pain and its association with sociodemographic, clinical and behavioral/psychosocial indicators among 8- to 10-year-old Brazilian schoolchildren.

## Materials and methods

### Ethical issues

The present study was carried out in compliance with international statutes and national legislation on ethics in research involving human subjects. All children assented to participation, and their parents or guardians signed an informed consent form. The present study received approval from the Human Research Ethics Committee of the Federal University of Santa Catarina (process n° 343.658/2014).

### Study design and sample size calculation

A cross-sectional study was carried out with children randomly selected from public schools of Florianopolis, Brazil from May to December 2015. Florianopolis is divided into twelve administrative districts and comprises 120 municipal schools, which included 16,234 students enrolled in primary education in 2015 [13].

To ensure representativeness, sample distribution was proportional to the total population enrolled in public schools for each of twelve administrative districts of the city. The subjects were randomly selected using a two-stage sampling method. The first stage was the randomization of public schools in each administrative district and the second was the randomization of the classrooms. All 8- to 10-year-old children belonging to the selected classrooms were invited to participate.

In order to allow the comparison of the results between different age groups, the strategy of uniform stratified sample selection was used. The prevalence of dental pain was considered 50%, since it was unknown in this population, a 95% confidence interval (CI) and standard error of 5% were adopted, which resulted in 354 participants in each age group, for a minimum sample size of 1,062 children. A correction factor of 1.2 was applied to increase the precision, since a multistage sampling method was adopted [14], resulting in a sample of 1,275 children. Next, 20% was added to compensate for possible losses, finally resulting in the recruitment of a total of 1,530 children.

To be included in the study, the children had to be 8 to 10 years of age, regularly enrolled in school, and accompanied by a Brazilian Portuguese-language speaking caregiver. Children with diseases that affected the central nervous system and those who were taking medications that could interfere with the central nervous system were excluded from the study due to the need to understand the applied questionnaires. Children undergoing orthodontic treatment were also excluded.

### Calibration exercise and pilot study

Prior to data collection, four examiners and four assistants were trained. Initially, examiners received theoretical training in a weekly range regarding the types of oral disorders (dental caries and its clinical consequences–pufa, dental fluorosis, dental erosion, dental trauma and molar incisor hypomineralization [MIH]) and their respective classification indexes and discussed clinical cases. The assistants were trained with the purpose of standardizing the annotation method. Any divergences were discussed and resolved by consensus. Subsequently, a calibration between the examiners and the gold standard was carried out with an oral examination of 20 children aged eight to 10 years. The calibration exercise comprised a period of 14 days. Initially, children were examined by the gold standard and the four examiners independently. After 7 days, the same children were examined again to assess the stability of the examiners to accurately diagnose the dental conditions and the intra-examiner and inter-examiner Kappa value were obtained. If necessary, a third examination would be conducted within 14 days of the first examination. Intra-examiner Kappa values for the DMFT/dmft, PUFA/pufa, MIH, fluorosis, dental erosion and dental trauma were >0.75 and the inter-examiner values were >0.70.

After the calibration process, a pilot study was conducted with 33 children from a public school and their caregivers. The objective of the pilot study was to assess the methodology reproducibility, which included the administration of a questionnaire containing behavioral and psychosocial indicators and sociodemographic questions. Furthermore, a clinical oral examination was also conducted in order to verify the criteria’s reliability. The participants of the pilot study were not included in the main study and no methodological changes were necessary since the instruments were considered appropriate for the study purpose.

### Non-clinical data collection

Non-clinical data collection directed to the children was performed in the school environment before the clinical oral examination. Information about the presence of dental pain was collected through a question directed to the children, as follows: “In the last month, how many times have you had pain in your teeth?”. Next, the variable was dichotomized as absent pain (answer “not once”) and present pain (answer “once or more”).

Information about behavioral and psychosocial indicators was collected through questions from the Brazilian version of the Child Perception Questionnaire 8–10 years (CPQ_8–10_) [15]. The term ‘psychosocial indicator’ is usually used as an umbrella term that is defined as the interrelation between social factors and individual’s mind in influencing behaviors, health, and wellbeing [16]. In the present study, the term “behavioral and psychosocial indicators” refers to the variables usually related to oral health impacts on daily living (trouble sleeping, difficulty eating, school absenteeism, difficulty paying attention in class, difficulty doing homework and staying away from recreational activities). If children answered “I don’t know” to any question, their caregiver was contacted.

The socioeconomic status and demographic conditions such as caregiver’s education (years of study) and household income (categorized based on the Brazilian monthly minimum wage [approximately U$ 225 during the data gathering]) were obtained through a questionnaire sent to the caregivers. Caregivers’ education was categorized based on a cut-off point of 8 years, which corresponds to a primary school education in Brazil. In addition, two questions about traumatic dental injuries (TDI) were included in the caregiver’s questionnaire to aid in the diagnosis and record the percentage of reported trauma in the primary and permanent dentitions.

### Clinical oral examination

Clinical data collection was conducted in the school environment. The procedures complied with biosafety norms. Each child was placed seated in a chair, the oral cavity was cleaned with gauze and, with the aid of an artificial light (Petzl Zoom head lamp; Petzl America, Clearfield, UT, USA) and #5 mouth mirrors (PRISMA, São Paulo, SP, Brazil), the visual inspection was performed. To verify the caries experience in the permanent and primary teeth, the DMFT/dmft index was used [17]. The DMFT/dmft expressed the total number of teeth [T/t] that were decayed [D/d], missing [M/m], or filled [F/f] in each child. When a carious lesion(s) or both carious lesion(s) and a restoration were present, the tooth was recorded as a D/d. When a tooth had been extracted due to dental caries, it was recorded as a M/m. Teeth restored for reasons other than dental caries were not counted as an F [17]. Caries severity was evaluated using a classification proposed by Feldens et al. [18], as follows: caries free (DMFT/dmft = 0), low severity (DMFT/dmft = 1–3) and high severity (DMFT/dmft >3). Untreated caries severity was determined isolating the “decayed” component of the DMFT index, as follows: caries free (D/d = 0), low severity (D/d = 1–3) and high severity (D/d >3). The clinical consequences of untreated caries lesions on permanent and primary teeth were measured by the PUFA/pufa index, which verified the presence of pulpal involvement [P/p]; ulceration of soft tissues due to tooth fragments from decayed crowns [U/u]; fistula [F/f]; and abscesses [A/a] [19]. Clinical information about TDI in the permanent anterior teeth was collected, following the classification proposed by Andreasen et al. [20]. Children were classified as with or without TDI. Dental fluorosis was accessed through the Dean Index [21]. MIH was diagnosed according to the criteria proposed by the European Academy of Pediatric Dentistry [22], which includes changes in at least one permanent first molar as: marked opacity greater than 2 mm; post-eruptive enamel fracture; and/or atypical restorations where there is presence of opacity in their margin. The children with hypoplastic spots only on the incisors were not considered carriers of MIH [22]. Dental erosion had been considered since the loss of superficial enamel characteristics, dentin exposure, wear on dentin and even pulp exposure due to wear. The permanent teeth were not evaluated for the presence of dental erosion due to the small period erupted in the buccal cavity. Dental fluorosis, dental erosion and molar-incisor hypomineralization [23,24,25] can cause pain in severe cases, in which the exposure of dentine or even the exposure of the pulp due to dental frangibility can occur, thus leading to dental sensitivity or dental pain. Therefore, these conditions were evaluated as possible confounding factors of dental pain.

### Data analysis

The statistical analysis were performed in the Statistical Package for Social Sciences (SPSS for Windows, version 21.0, SPSS Inc. Chicago, IL, USA) program. Initially, descriptive analysis were performed. To verify the association between dental pain with sociodemographic and clinical variables the question about the presence of dental pain was the dependent variable. The chi-square and Fisher’s exact tests were conducted to determine associations between the dependent and independent variables. Next, a hierarchical approach to select variables was used [26]. The variables were grouped into a hierarchy of categories ranging from distal to proximal determinants for the occurrence of dental pain. The categories were included in this order: sociodemographic factors and clinical conditions. For each level, a Poisson regression analysis with robust variance was performed This analysis was performed to exclude variables with a P-value > 0.20. Explanatory variables were selected for the final models only if they had a P-value of < 0.05 after adjustment for variables on the same or previous levels of determinants. The prevalence ratios (PR) and 95% confidence intervals were calculated. To verify the association between dental pain and behavioral/psychosocial indicators, dental pain was considered the predictor. For each behavioral/psychosocial indicator, a Poisson regression analysis with robust variance was performed, considering those as dependent variables. Prevalence ratios (PR) and 95% confidence intervals were also calculated.

## Results

A total of 1,589 children (mean age 8.9 ± 0.8 years old) participated in the study (~42% boys and 58% girls). The response rate was 89.5% and the missing data corresponded to unanswered questionnaires. The proportion of children that reported episodes of dental pain in the previous four weeks was 51.5% (n = 819).

Table 1 presents the frequency distribution and bivariate analysis of sociodemographic factors and clinical conditions on the presence of dental pain. Independent variables such as sex, self-reported dental trauma, caries experience, caries severity, untreated caries severity, PUFA/pufa index, pulpal involvement and fistula were significantly associated with the presence of dental pain (p<0.05). Caregivers’ education, ulceration and abscess were also included in the Poisson regression model (p<0.20).

**Table 1:** Frequency distribution and bivariate analyses of socioeconomic factors, individual variables and clinical conditions on the presence of dental pain in schoolchildren (n = 1589).

Independent variables	n (%)	Dental Pain		P*
		No n (%)	Yes n (%)	
**Sociodemographic variables**				
Sex				**< 0.001**^a^
Female	916 (57.6)	407 (44.4)	509 (55.6)	
Male	673 (42.4)	363 (53.9)	310 (46.1)	
Age				0.626^b^
8 years-old	533 (33.5)	258 (48.4)	275 (51.6)	
9 years-old	570 (35.9)	269 (47.2)	301 (52.8)	
10 years-old	486 (30.6)	243 (50.0)	243 (50.0)	
Caregiver’s education				0.078^a^
≤ 8 years	453 (30.6)	206 (45.5)	247 (54.5)	
> 8 years	1029 (69.4)	519 (50.4)	510 (49.6)	
Household income				0.611^a^
≤ 2 BMW*	752 (52.4)	356 (47.3)	396 (52.7)	
> 2 BMW	684 (47.6)	333 (48.7)	351 (51.3)	
**Clinical conditions**				
Caries experience				**< 0.001**^**a**^
dmft/DMFT = 0	686 (43.2)	394 (57.4)	292 (42.6)	
dmft/DMFT ≥ 1	903 (56.8)	376 (41.6)	527 (58.4)	
Caries severity				**< 0.001**^b^
Caries free (dmft/DMFT = 0)	686 (43.2)	394 (57.4)	292 (42.6)	
Low severity (dmft/DMFT 1–3)	597 (37.6)	266 (44.6)	331 (55.4)	
High severity (dmft/DMFT ≥ 3)	306 (19.3)	110 (35.9)	196 (64.1)	
Untreated caries severity				**< 0.001**^b^
Caries free (D/d-dmft/DMFT = 0)	898 (56.5)	500 (55.7)	398 (44.3)	
Low severity (D/d-dmft/DMFT 1–3)	536 (33.7)	219 (40.9)	317 (59.1)	
High severity (D/d-dmft/DMFT ≥ 3)	155 (9.8)	51 (32.9)	104 (67.1)	
PUFA/pufa index				**< 0.001**^**a**^
PUFA/pufa = 0	1244 (78.3)	652 (52.4)	592 (47.6)	
PUFA/pufa ≥ 1	345 (21.7)	118 (34.2)	227 (65.8)	
Pulpal involvement (P/p)				**< 0.001**^**a**^
No	1373 (86.4)	701 (51.1)	672 (48.9)	
Yes	216 (13.6)	69 (31.9)	147 (68.1)	
Ulceration (U/u)				0.125^c^
No	1582 (99.6)	769 (48.6)	813 (51.4)	
Yes	7 (0.04)	1 (14.3)	6 (85.7)	
Fistula (F/f)				**0.034**^**a**^
No	1561 (98.2)	762 (48.8)	799 (51.2)	
Yes	28 (1.8)	8 (28.6)	20 (71.4)	
Abscess (A/a)				0.068^a^
No	1558 (98.0)	760 (48.8)	798 (52.2)	
Yes	31 (2.0)	10 (32.3)	21 (67.7)	
Dental fluorosis				0.972^a^
Absent	1016 (63.9)	492 (48.4)	524 (51.6)	
Present	573 (36.1)	278 (48.5)	295 (51.5)	
Dental erosion				0.643^a^
Absent	1338 (84.2)	645 (48.2)	693 (51.8)	
Present	251 (15.8)	125 (49.8)	126 (50.2)	
Self-reported dental trauma				**0.015**^**a**^
No	1125 (73.2)	566 (50.3)	559 (49.7)	
Yes	411 (26.8)	178 (43.3)	233 (56.7)	
Clinical sign of dental trauma				0.795^a^
Absent	1421 (89.4)	687 (48.3)	734 (51.7)	
Present	168 (10.6)	83 (49.4)	85 (50.6)	
MIH**				0.710^a^
Absent	1438 (90.5)	699 (48.6)	739 (51.4)	
Present	151 (9.5)	71 (47.0)	80 (53.0)	

^a^ Chi-squared test (P < 0.05)

^b^ Linear-by-linear association

^c^ Fisher's exact test

* BMW: Brazilian minimum wage (approximately U$ 225 during the data gathering)

**MIH: Molar-incisor hypomineralization

The results of the unadjusted and adjusted regression analysis for sociodemographic and clinical factors associated with dental pain reported in the last month are shown in Table 2. The variables were incorporated in the Poisson regression model and those with p<0.05 on the non adjusted analysis were incorporated on the adjusted model. Following the adjustment, it was revealed that sex, caries experience, PUFA/pufa index, and ulceration remained statistically significant (p<0.05).

**Table 2:** Final Poisson regression model for covariates associated with dental pain in schoolchildren (n = 1589).

Independent variables	Unadjusted PR (95% CI)	P	Adjusted PR (95% CI)	P
**Sociodemographic variables**				
Sex				
Male	1		1	
Female	1.16 (1.04–1.29)	**0.006**	1.18 (1.07–1.31)	**0.001**
Caregiver’s education				
≤ 8 years	1			
> 8 years	0.90 (0.81–1.00)	0.052	-	**-**
**Clinical conditions**				
Caries experience				
dmft/DMFT = 0	1		1	
dmft/DMFT ≥ 1	1.36 (1.22–1.51)	**< 0.001**	1.30 (1.16–1.45)	**<0.001**
Caries severity				
Caries free (dmft/DMFT = 0)	1			
Low severity (dmft/DMFT 1–3)	1.29 (1.15–1.45)	**< 0.001**		
High severity (dmft/DMFT > 3)	1.49 (1.31–1.69)	**< 0.001**	-	-
Untreated caries severity				
Caries free (D/d-dmft/DMFT = 0)	1			
Low severity (D/d-dmft/DMFT 1–3)	1.29 (1.16–1.44)	**< 0.001**		
High severity (D/d-dmft/DMFT > 3)	1.52 (1.33–1.74)	**< 0.001**	-	-
PUFA/pufa index				
PUFA/pufa = 0	1		1	
PUFA/pufa ≥ 1	1.37 (1.23–1.52)	**< 0.001**	1.24 (1.12–1.39)	**<0.001**
Pulpal involvement (P/p)				
No	1			
Yes	1.36 (1.21–1.53)	**< 0.001**	-	-
Ulceration (U/u)				
No	1		1	
Yes	1.68 (1.24–2.29)	**0.001**	1.33 (1.01–1.75)	**0.046**
Fistula (F/f)				
No	1			
Yes	1.31 (0.98–1.75)	0.065	-	-
Abscess (A/a)				
No	1			
Yes	1.31 (1.00–1.72)	0.050	-	-
Self-reported dental trauma				
No	1			
Yes	1.12 (1.01–1.25)	0.032	-	-

RR: Robust Rate Ratio; CI: Confidence Interval

Table 3 presents the results of the unadjusted and adjusted Poisson regression analysis for behavioral/psychosocial indicators associated with dental pain. After adjustment with sociodemographic and clinical variables, dental pain was considered a predictor of all behavioral/psychosocial indicators investigated in the present study (p<0.05).

**Table 3:** Poisson regression model for association between behavioral/psychosocial indicators and dental pain in schoolchildren (n = 1589).

	Unadjusted PR (95% CI)	P	Adjusted PR (95% CI)	P
	**Trouble Sleeping**			
Dental Pain^a^				
Absent	1		1	
Present	3.05 (2.48–3.75)	**<0.001**	2.87 (2.34–3.51)	**<0.001**
	**Difficulty Eating**			
Dental Pain^b^				
Absent	1		1	
Present	2.35 (2.02–2.74)	**< 0.001**	2.28 (1.96–2.66)	**< 0.001**
	**School Absenteeism**			
Dental Pain^c^				
Absent	1		1	
Present	2.48 (1.97–3.12)	**< 0.001**	2.29 (1.83–2.85)	**< 0.001**
	**Difficulty with paying attention in class**			
Dental Pain^d^				
Absent	1		1	
Present	3.35 (2.60–4.33)	**< 0.001**	3.47 (2.64–5.30)	**<0.001**
	**Difficulty with doing homework**			
Dental Pain^e^				
Absent	1		1	
Present	3.90 (2.73–5.59)	**< 0.001**	1.23 (1.11–1.35)	**< 0.001**
	**Staying away from recreation activities**			
Dental pain^f^				
Absent	1		1	
Present	1.72 (1.57–1.89)	**< 0.001**	3.18 (2.22–4.55)	**<0.001**

^a^Adjusted for untreated dental caries and PUFA index.

^b^Adjusted for caries severity.

^c^Adjusted for age and caries experience.

^d^Adjusted for sex, caries severity and dental fluorosis.

^e^Adjusted for age, caregiver’s education and caries experience.

^f^Adjusted for caries severity, PUFA index and dental fluorosis.

## Discussion

The present cross-sectional survey assessed the prevalence of self-reported dental pain and its associated factors in a sample of 8- to 10- year-old schoolchildren from South Brazil. Dental pain is considered a public health problem due to its negative impacts on society in means of high costs of treatment, absenteeism from school, the use of medications [10] and mainly on children’s emotional and social well-being. In this sense, the ages of 8–10 years are of pivotal importance in the psychological development. For example, between 8 to 10 years old, children begin to develop the ability to judge their emotions and behavior, as well as the thoughts of others regarding their physical appearance [27]. Therefore, these factors motivated the development of the present manuscript.

In the present study, a prevalence of self-reported dental pain of 51.5% was found. A considerably lower prevalence of dental pain (7.6%) was observed in 8-year-old schoolchildren from England [3] and Brazil [7], whereas the prevalence of dental pain in the previous month to the study was 15.6% in 8- to 9-year-old schoolchildren. This difference can be sustained by the methodological rigor of the present study. A representative sample, randomly selected from schools was included and the prevalence of dental pain was obtained by self-reports of children with a high response rate. It is also important to point out that the population included in the research was in the mixed dentition (8–10 years old). At this age, the child has several teeth in the period of exfoliation and, therefore, with mobility. This increased mobility may cause some discomfort [10], which could have interfered with children’s answers.

In the current study, the presence of dental pain was more frequent in girls. This finding has also been described in studies with preschool children [9], schoolchildren [28,29] and adolescents [12]. This difference between sexes possibly reflects social norms, values and the establishment of cultural rules and patterns, in which boys should express less pain and, consequently, reported less dental pain [29]. Therefore, preventive care consultations are very important for the early diagnosis of possible clinical indicators of dental pain, since many of those boys may experience dental pain and may have a higher resistance of expressing their symptoms and feelings [28].

Some epidemiological surveys have established associations between dental pain and socioeconomic indicators such as caregiver’s schooling and family income [11,12,30], whilst children of parents with low educational level and lower socioeconomic status tend to experience more dental caries and, consequently, more dental pain. However, this association was not found in the present study. This difference can be explained by the characteristics of the sample. The educational level and family income in the present study were higher when compared with other populations, which reflects the reality of the state of Santa Catarina, Brazil, which holds the third highest Human Development Index and fourth Income Index in Brazil [31].

Caries experience is one of the most common causes of dental pain in children [11,30]. In the present study, caries experience was significantly associated with dental pain, whereas children with at least one decayed, missed or filled tooth reported more dental pain. However, the reports of dental pain were not associated with caries experience severity nor untreated dental caries severity. Thereby, these results showed that the presence of dental caries by itself is sufficient to cause dental pain, while the quantity of lesions, translated as severity, did not show an association. Therefore, the present study demonstrates the importance of preventing rather than only treating the consequences of dental caries. Moreover, clinical consequences of untreated dental caries, determined by the PUFA index, were also significantly associated with dental pain, in accordance with a previous study [9]. When evaluating each component of the PUFA index separately, only the presence of ulceration remained significant. This result should be interpreted with caution since the prevalence of ulceration was very low (only 7 cases), and therefore further studies are necessary to clarify this association.

In the current study, other clinical conditions were evaluated, such as dental fluorosis, dental erosion, dental trauma and molar-incisor hypomineralization, although no association with dental pain was found. These conditions can cause pain only in severe cases, in which the exposure of dentine or the exposure of the pulp due to dental frangibility leads to dental sensitivity or dental pain [23,24,25]. However, in the sample, severe cases of these clinical conditions were not frequently observed. Therefore, further studies should be conducted considering children with severe cases of dental fluorosis, dental erosion, dental trauma and/or molar-incisor hypomineralization to evaluate their association with dental pain.

The association between dental pain and psychosocial indicators such as difficulty sleeping, difficulty eating, school absenteeism, difficulty with paying attention in class, difficulty doing homework and staying away from recreational activities was found in the present research. These results highlight the importance of preventing dental pain episodes in children since several hormones important to children’s development are involved with sleep or are released during sleep, such as the growth hormone [32]. Furthermore, short sleep can result in extended hours of wakefulness, leading to opportunities to increase food intake typically in the form of convenient carbohydrate-rich and energy-dense snacks [33].

Among children with history of dental pain, 71.6% reported difficulty eating. The presence and severity of dental caries have been associated to dental pain and problems in eating certain types of food [10,11]. Furthermore, studies have found that parents of children with severe dental caries reported a greater impact on the OHRQoL of their children, namely more pain and a decreased ability to chew [34]. This finding could be explained by considering that untreated dental caries and associated infection could cause pain, discomfort and reduce intake of foods since eating could be painful [35]. Moreover, the associations between dental pain, school absenteeism, difficulty with paying attention in class and difficulty doing homework are very important findings considering the fundamental role of the school on the development of children as citizens. Dental pain can exert negative consequences not only in the learning process, but also in other important aspects such as socialization [34]. Moreover, considering the adult life, the lack of education can influence the capacitation of the individual as a professional and, consequently, hamper insertion in the employment market, which can reflect on the economy of a country.

The association between dental pain and staying away from recreational activities may be explained by the bullying these children with dental pain may experience. Children aged 8 to 10 years-old with dental caries reported suffering verbal bullying [36], and since dental caries is still the main cause of occurrence of dental pain, those children with dental pain may as well have difficulty socializing with other children [34] and may experience bullying.

The strengths of the present study should be highlighted. First, a representative sample with a high response rate was obtained, conferring credibility and reliability to the present research. Beyond that, few studies seeking to determine dental pain prevalence have been conducted in this age group, highlighting its originality and importance to the field, since children aged 8- to 10-years are in a pivotal phase of growth and development. Furthermore, a large number of clinical variables were investigated in this study. Although many studies have reported the association between dental pain and dental caries, none has tested the association between dental pain and other clinical conditions, such as dental fluorosis, dental erosion and MIH. Some limitations should also be discussed. The cross-sectional design does not give the time relationship between the variables, which does not allow determining causality between the predictors and the outcome. Also, children’s experience of pain was assessed by a single question, not a specific validated questionnaire, although reliable responses were obtained from children, since pain is a subjective phenomenon that can be measured by self-reported questions [37]. Moreover, only children from public schools were included in the present study, therefore, conclusions should be made carefully.

This present study evidenced the role of dental caries experience and its clinical consequences on dental pain, and how this can affect the daily living of children, impairing important aspects of their life such as growth, development, learning and socialization. Pain is generally considered as a public health problem and dental pain is classified as a dental emergency that can indicate the effectiveness and efficiency of health services [25]. Therefore, the assessment of the prevalence of dental pain and its associated factors provides relevant information regarding the population needs and it should guide the implementation of health care policies, as well as preventive measures to assist children and their families to maintain good oral health since it is directly related to general well being.

## Conclusion

The present study indicated that the prevalence of self-reported dental pain in 8- to 10- year-old Brazilian schoolchildren was high. Sociodemographic, clinical and behavioral/psychosocial indicators such as sex, dental caries experience and its clinical consequences (PUFA index), school absenteeism and trouble sleeping were associated with the occurrence of dental pain. Thus, these findings make an important contribution to clinical decision-making and the establishment of preventive and treatment priorities in individual and public oral health care.
